# Transcriptomic Crosstalk between Fungal Invasive Pathogens and Their Host Cells: Opportunities and Challenges for Next-Generation Sequencing Methods

**DOI:** 10.3390/jof2010007

**Published:** 2016-01-14

**Authors:** Francisco J. Enguita, Marina C. Costa, Ana Marisa Fusco-Almeida, Maria José Mendes-Giannini, Ana Lúcia Leitão

**Affiliations:** 1 Faculdade de Medicina, Universidade de Lisboa, Av. Professor Egas Moniz, Lisboa 1649-028, Portugal; marinacosta@medicina.ulisboa.pt; 2 Núcleo de Proteômica, Faculdade de Ciências Farmacêuticas, Universidade Estadual Paulista—UNESP, Rodovia Araraquara—Jaú Km 1, Araraquara 14801-902, São Paulo, Brazil; ana.marisa@uol.com.br (A.M.F.-A.); gianninimj@gmail.com (M.J.M.-G.); 3 MEtRICs, Departamento de Ciências e Tecnologia da Biomassa, Faculdade de Ciências e Tecnologia, Universidade NOVA de Lisboa, Campus de Caparica, Caparica 2829-516, Portugal

**Keywords:** fungal pathogen, invasive infection, high-throughput transcriptomics, next generation sequencing, non-coding RNAs, dual RNA-seq

## Abstract

Fungal invasive infections are an increasing health problem. The intrinsic complexity of pathogenic fungi and the unmet clinical need for new and more effective treatments requires a detailed knowledge of the infection process. During infection, fungal pathogens are able to trigger a specific transcriptional program in their host cells. The detailed knowledge of this transcriptional program will allow for a better understanding of the infection process and consequently will help in the future design of more efficient therapeutic strategies. Simultaneous transcriptomic studies of pathogen and host by high-throughput sequencing (dual RNA-seq) is an unbiased protocol to understand the intricate regulatory networks underlying the infectious process. This protocol is starting to be applied to the study of the interactions between fungal pathogens and their hosts. To date, our knowledge of the molecular basis of infection for fungal pathogens is still very limited, and the putative role of regulatory players such as non-coding RNAs or epigenetic factors remains elusive. The wider application of high-throughput transcriptomics in the near future will help to understand the fungal mechanisms for colonization and survival, as well as to characterize the molecular responses of the host cell against a fungal infection.

## 1. Introduction

Fungi are a diverse and complex group of eukaryotic microorganisms present in the environment that can be part of the microflora of animals and plants. Some fungal species have the ability to produce infections ranging from mild superficial colonization to severe life-threatening conditions. Invasive fungal infections (IFIs) are described by the presence of fungi colonizing deep tissues. In humans, these infections have an increasing clinical importance, empowered by the growing number of cases observed in the last decade. Fungal systemic infections are normally rare, but their mortality rates are very high when compared with bacterial and viral infections [1]. Interestingly, in systemic fungal infections, the outcome of the disease depends more on the host factors than on the intrinsic fungal virulence [2]. Fungal infections are a major health problem, mainly related with the immunosuppression associated with conditions such as human immunodeficiency virus (HIV) infection, cytomegalovirus (CMV) infection, tuberculosis, and transplantation, among others. In plants, fungal infections are also a significant threat to the economy and public health, since some species are able not only to cause localized and systemic infections, but also to produce allergens and toxins that can contribute to respiratory diseases and allergies [3].

Fungal infections are very complex processes that evolve from the initial contact of the infectious agent with the host surface, the colonization and spreading of the infective cells, and the final establishment of the infection in its target organ. The infection progress and outcome depends on several pathogen and host factors, including the immunological status of the patients, localization of the infected area, and the production of fungal virulence factors. Infectious agents of fungal origin are prone to produce diverse factors that facilitate the interaction and colonization of the host. In the majority of cases, those virulence factors are complex and multifactorial and can be classified into two categories: those that promote colonization and invasion, and those causing direct harm to the host, such as toxins and extracellular enzymes [4]. Host response against the infection is also extremely relevant for IFIs, involving either innate or adaptive immune responses [5,6]. Interestingly, IFIs are considered as a paradigm in immunology as they can arise either from the lack of recognition of the pathogen by the immune system or by an exaggerated inflammatory response [5].

Prevention of a fungal disease depends on a deeper knowledge of the etiological agent, its virulence factors, and its interaction with the host. Fungal infections can be considered as changes in the natural life cycle of certain fungi, which in most cases require specific host determinants that allow its development within the cells [7,8]. These infections have attracted increasing attention from the scientific community, due to the great inefficiency in the treatment of their systemic forms. The overall knowledge of the molecular mechanisms governing the interactions between fungal cells and their host is still in its infancy. A lot of efforts have been made to characterize the fungal determinants of infection, disregarding the host factors to a second plane [4,9]. As a consequence, there is an unmet need to understand the communication channels between host cells and infective fungi during the process of interaction and colonization. A possible approach will be to characterize the transcriptional cross-talk triggered by the infective fungi and their host cells that could lead to the design of more effective therapeutic strategies. High-throughput transcriptomic analysis by next-generation sequencing (RNA-seq) in all its possible flavors (mRNA-seq, small-RNA-seq, dual RNA-seq, among others), is becoming a method of choice for the integrative analysis of communication networks between fungal pathogens and their host-cells.

## 2. Expression of Virulence Factors and Transcriptional Landscape of Infection in Fungal Pathogens

### 2.1. Invasive Fungi Causing Infections in Humans

The variety of fungi comprises 1.5 million species, of which approximately 7400 are characterized. Among them only a few are pathogenic to humans, belonging to four main groups: zygomycetes, ascomycetes, deuteromycetes, and basiomycetes. The growth of highly lethal opportunistic fungal infections in recent decades, including invasive mycoses, is attributed mainly to an increased number of patients with severe immunosuppression [10,11]. On the other hand, another aggravating factor is related with the presence of resistant or less susceptible strains to the currently available antifungal agents [12,13]. Deaths caused by systemic mycoses such as paracoccidioidomycosis, cryptococcosis, histoplasmosis, candidiasis, aspergillosis, coccidioidomycosis and zygomycosis have recently reached quite significant numbers. Among the pathogens that cause invasive fungal infections, *Candida* and *Cryptococcus* species are predominant in immunocompromised individuals [1,14]. Host colonization is achieved by transcriptional programs of virulence genes controlled by groups of specific transcription factors [15]. The emergence of high-throughput techniques of transcriptional analysis in recent years, namely RNA-seq, is starting to be employed for unraveling the specific molecular details of these transcriptional events in pathogenic fungi (See Table 1).

*Candida albicans* is probably the most versatile opportunistic fungal pathogen. It belongs to the normal commensal microflora, but under favorable conditions it can cause a panoply of infections, ranging from superficial mucosal colonization to a systemic infection [16]. As dimorphic fungi, *Candida* species showed phase transition between yeast and filamentous structures, and this transition is a key factor in the pathogenesis [17]. RNA-seq experiments have determined that pathogenic *C. albicans* causing vaginal infections in mice show a specific overexpression of hypha-associated secreted enzymes, mainly aspartyl-proteinases 4, 5, and 6 (SAP4–6), which are known inflammasome activators [18]. This gene expression pattern appears to be general and independent of the site of infection, as demonstrated also in systemic infections [19].

**Table 1:** Selected next generation sequencing datasets containing transcriptomic data (RNA-seq) from fungal infections in mammalian cells.

Database	Access Code	Subject	NGS Platform	Reference
GEO	GSE55663	Transcriptomic analysis of antifungal activity by humidimycin over *Aspergillus fumigatus*	Illumina HiSeq 2000	[20]
GEO	GSE32049 GSE32228	Transcriptomic analysis of capsule regulation in *Cryptococcus neoformans* strains	Illumina Genome Analyzer IIx and Illumina HiSeq 2000	[21]
GEO	GSE40425	Transcriptomic analysis of the response of *Tricophyton rubrun* to acriflavine	AB SOLiD 4 System	[22]
GEO	GSE43189	*Cryptococcus neoformans* gene expression associated with cell wall remodeling and evasion of the immune system	Illumina Genome Analyzer II	[23]
GEO	GSE43363	RNAi-mediated genomic defense in *Cryptococcus neoformans*	Illumina Genome Analyzer II	[24]
GEO	GSE51573	*Cryptococcus* *neoformans* transcriptome analysis at the site of human meningitis	Illumina HiSeq 2000	[25]
GEO	GSE56091	Characterization of transcriptome dynamics of *Candida albicans* in response to contact with host cells	Illumina HiSeq 2000	[26]
GEO	GSE57217	Cross talk between the cell wall integrity and cAMP/protein kinase A pathways in *Cryptococcus neoformans*	Illumina HiSeq 2000	[9]
GEO	GSE60398	Virulence regulation in *Cryptococcus neoformans*	Illumina HiSeq 2000 and Illumina Hiseq 2500	[15]
GEO	GSE61550	Epigenetic regulation of virulence and genomic specificity in *Cryptococcus neoformans*	Illumina HiSeq 2500	[27]
GEO	GSE67688	Transcriptomic analysis of vulvovaginal candidiasis in mouse	Illumina HiSeq 2000	[18]
GEO	GSE70227	*Aspergillus fumigatus* in blood infections	Illumina HiSeq 2000	[28]
SRA	SRP055976	Transcriptomic analysis of *Pseudogymnoascus destructans* infection in bats	Illumina HiSeq 2500	[29]
SRA	SRP058281	*Candida albicans* transcriptome during infection of mouse kidneys and *Galleria mellonella*	Illumina HiSeq 2500	[30]
SRA	SRP028588	*Histoplasma capsulatum* yeast and mycelia transcriptomes	Illumina Genome Analyzer II	[31]

*Cryptococci* are dimorphic fungi that produce respiratory infections through inhalation of infectious particles (basidiospores) or desiccated yeasts present in the environment, which, thereafter colonize the alveolar tissue [32]. In lung tissue they are able to remain in a latency stage or manifest themselves by variable signs and symptoms, which can vary from asymptomatic patients to cases of severe pneumonia and respiratory failure [33]. In healthy individuals, the infection is effectively counteracted by pro-inflammatory immune T-cell response, but in immunocompromised patients, the yeast spreads easily through the blood, colonizing various organs, the central nervous system (CNS) being its major target [33]. It is believed that one of the ways in which *Cryptococcus* reaches the CNS, crossing the blood–brain barrier, is within macrophages thanks to a specialized cell wall that allows the pathogen to remain undetected by the immune system [34,35]. One of the major *Cryptococcus* virulence factors is the Rim101 transcription factor which regulates the cell-wall composition at the host–pathogen interface, providing an effective stealth mask for the immune system [23]. The biosynthesis and structural properties of the cell wall is controlled by the cell-wall integrity signaling pathway (CWI), which involves at least four different kinases, PKC1, BCK1, MKK2, and MPK1. Recent high-throughput analysis by next-generation sequencing has determined that the deletion of at least one of these kinases results in a differential gene expression pattern characterized by the predominance of genes regulated by cyclic-AMP [9].

Other pulmonary fungal pathogen of great importance is the dimorphic fungus *Histoplasma capsulatum*, causing histoplasmosis which starts by the inhalation of microconidia or hyphal fragments. After pulmonary infection and depending on the immune status of the host, these forms can spread to other organs belonging to the mononuclear phagocytic system, mainly the spleen and liver, causing the most severe form of histoplasmosis [36]. The role of cellular immunity in protection against *H. capsulatum* has been already described [37]. Thus, in immunocompromised patients, especially in HIV-positive ones, histoplasmosis is opportunistic, in most cases widespread and, if untreated, leads to death in almost 100% of cases [38,39]. *Histoplasma* dimorphism is characterized by the presence of avirulent mycelia and pathogenic yeast, the transition between these forms being an important virulence factor. Comparison of phase transcriptomes determined by RNA-seq revealed a small amount of differentially regulated transcripts between mycelia and a yeast phase (6% to 9%), which is comparatively similar to the proportion of differentially expressed transcripts between different pathogenic *H. capsulatum* strains [31]. Differentially expressed genes between mycelia and yeast cells showed virulence factors, but also genes encoding enzymes involved in protein glycosylation, energy metabolism, and cell wall maintenance [31]. Interestingly, RNA-seq experiments also revealed that *H. capsulatum* yeast showed a specific transcriptional fingerprint when submitted to physical stress such as hypoxia [40] or temperature [41], which could be related to phase transition phenomena and the intrinsic pathogenicity of this fungus.

Another fungus of extreme clinical relevance is *Paracoccidioides* spp., responsible for paracoccidioidomycosis. *Paracoccidioides* is a dimorphic fungus that growths naturally as a saprophytic mycelia. The transmissible forms of the fungus are constituted by its conidia, that when inhaled will originate the infective yeast cells within the host organism [42]. In this genus, the transcriptional reprogramming associated with phase transition and the responses against stress have started to be unraveled, but the use of high-throughput method is comparatively less extended [42,43].

### 2.2. Plant Pathogens

Fungal pathogens infecting plants have been an important subject of study because of their economical implications. Interestingly, the use of high-throughput techniques for transcriptome analysis in plant fungal pathogens has been comparatively more extended than in fungi infecting animal cells (Table 2) [44]. Plant fungal pathogens are extremely diverse in their genomes, pathogenic determinants, virulence, and physiology, and a detailed analysis of this topic is out of the scope of this review [16]. Because of their intrinsic importance, three plant pathogens can be considered as infection models: *Ustilago maydis*, an unicellular budding yeast belonging to the phylum Basidiomycota able to infect corn and other teosinte plants; *Fusarium oxysporum*, a filamentous Ascomycota that infects several crops; and *Magnaporthe oryzae*, also belonging to the phylum Ascomycota, and characterized by its filamentous growth and extended infection capacity that can affect diverse monocotylous plants as rice, barley and wheat [16].

In *U. maydis*, RNA-seq has been used to characterize functional genes related to cell polarization and filamentous growth, but not within the context of infection [45]. On the other hand, transcriptome analysis by RNA-seq of different *F. oxysporum* strains disclosed a significant difference in transcriptional responses between them post-inoculation to a host plant in comparison to the vegetative growth stage, involving genes related to protein-G signaling pathways, mitogen-activated protein kinases, and specific membrane transport systems [46]. Indeed, among the model plant pathogens, the most widely studied strain is *M. oryzae* due to its intrinsic characteristics that makes it the most destructive pathogen affecting rice. Transcriptomic studies using simultaneous RNA-seq of the pathogen and host have been performed in *M. oryzae*, showing upregulation of fungal transcripts encoding glycosyl hydrolases, cutinases and LysM domain-containing proteins during infection [47]. Recently, RNA-seq has been employed to characterize hypoxia-responsive genes of *M. oryzae*, which could be involved in the establishment of the infection. Null mutants of selected hypoxia-responsive genes involved in sterol metabolism exhibited increased conidiation, and delayed invasive growth within host cells, which is suggestive of important roles in fungal development [48].

**Table 2:** Selected next-generation sequencing datasets containing transcriptomic data (RNA-seq) from fungal infections in plant cells.

Database	Access Code	Subject	NGS Platform	Reference
GEO	GSE32010	Genome-wide analysis of rice transcriptional change during the early stages of false smut formation caused by *Ustilaginoidea virens*	Illumina HiSeq 2000	[49]
GEO	GSE40952	Digital gene expression analysis of early root infection of *Sporisorium reilianum* f.sp. *zeae* in maize	Illumina Genome Analyzer	[50]
GEO	GSE57857	Small RNA-seq identification and characterization the microRNA response from chickpea against *Fusarium oxysporum* f.sp. *ciceris* infection	Illumina Genome Analyzer IIx	[51]
GEO	GSE67191	Comparative transcriptomics of Central Asian *Vitis vinifera* to characterize the distinct defense strategies against powdery mildew	Illumina HiSeq 2500	[52]
GEO	GSE57587 GSE57586	Transcriptional analysis of *Botrytis cinerea* targeting cells from grape and tomato	Illumina HiSeq 2000	[53]
GEO	GSE58653	Transcriptome analysis of fungal pathogen *Neofusicoccum parvum* in grapevine leaves	Illumina HiSeq 2500	[54]
GEO	GSE58958	Genomic structural variation in the grape mildew pathogen *Erysiphe necator*	Illumina HiSeq 2500	[55]
GEO	GSE40581	Transcriptome analysis of several pathogenic strains of *Fusarium oxysporum*	Illumina HiSeq 2000	[46]
GEO	GSE51597	Transcriptomic analysis of hypoxia-responsive genes in *Magnaporthe oryzae*	Illumina HiSeq 2000	[48]
SRA	SRP035525	Transcriptional analysis of *Brassica napus* infected by two *Leptosphaeria* species	Illumina HiSeq 2000	[56]
SRA	SRP015912	Transcriptome analysis of *Lactuca sativa* cv. *Salinas* after inoculation with *Botrytis cinerea*	Illumina HiSeq 2000	[57]
SRA	SRP052276	Response of *Arabidopsis thaliana* to *Fusarium oxysporum*	Illumina HiSeq 2000	[58]

## 3. Transcriptional Programs of the Host Cells in Response to a Fungal Infection

The host response against pathogenic infections is initially started by inflammatory and immune responses with the objective of the clearance of the pathogenic agent. On the other hand, the pathogenic agent has been evolved to reshape the host cell function to its own convenience, to promote survival and to generate a proper environment for the colonization of the host organism. The intricate networks involving host–pathogen interaction is not limited to the initial contact between both cells. Signaling pathways are activated upon infection, being linked to specific remodeling of the host cell transcriptional state in response to the presence of the pathogenic organism [59]. In some cases, the triggered transcriptional program is mainly composed of genes involved in innate immunity, but in the majority of occasions the host cell responds also in a pathogen-specific fashion [60]. Fungal-infected cells typically trigger an acute inflammatory response mediated by cytokines, including interleukins and chemokines, but the interactions between fungal virulence factors and signaling pathways within the host cell would modulate the progression of the infection in a specific manner. The overall understanding of this transcriptional response will enlarge our knowledge of the biology of host–pathogen interaction, potentially deriving new therapeutic targets and strategies, and also providing biomarkers to follow the progression of the infection and the results of a therapeutic intervention.

### 3.1. Human and Mammalian Host Cells

Most of the available data on the specific host-cell transcriptomic changes upon infection have been collected from bacterial and viral infections [61,62]. The transcriptomic response of animal cells to fungal pathogens has been also studied in a selected number of cases (Table 1). In an original pioneer work based on transcriptomic analysis by microarrays in *Aspergillus nidulans* conidia infecting airway epithelial cells, Oosthuizen and coworkers demonstrated the up-regulation of interleukin IL-6 along with several other host genes after their interaction. Interestingly, the host-cell transcriptomic changes induced by *A. nidulans* infection were cell specific, showing clear differences between primary cells and cell lines [63]. In *Candida albicans* infection of innate immune cells, next-generation experiments allowed detection and validation of several interaction networks between pathogen and host [64]. These specific interaction networks appeared to act together with the innate immune response against the infection. The authors proposed an integrated model for the functionality of these interaction networks during fungal invasion of immune cells, involving the binding of Ptx3 protein to the *C. albicans* cell wall and an induced remodeling via fungal Hap3 target genes, altering the immune response to the pathogen [64]. Very recently, the work by Field *et al.* [29] corroborated the observations already described in other fungal infections, such as those by *C. albicans*. In this study, the authors performed a transcriptomic analysis of American bats infected by psychrophilic fungus *Pseudogymnoascus destructans*, which causes a necrotic invasive cutaneous infection designated as “white-nose” syndrome. In cells infected by *P. destructans*, gene expression was increased for inflammatory cytokines, including interleukins (IL) IL-1β, IL-6, IL-17C, IL-20, IL-23A, IL-24, and G-CSF, and chemokines such as Ccl2 and Ccl20. This pattern of gene expression changes demonstrates that white-nose syndrome is accompanied by an innate anti-fungal host response similar to that caused by cutaneous *C. albicans* infections [29,64].

### 3.2. Plant Host Cells

In food crops, fungal pathogens frequently establish a pathogenic relationship with an initial colonization and necrosis that could eventually lead to plant death, resulting in serious economic losses. This is the case with *Botrytis cinerea*, a plant pathogen that can parasitize several crops, including lettuce (*Lactuca sativa*). Experimental approaches based on next-generation sequencing determined that the interaction between *B. cinerea* and *L. sativa* causes a pronounced decrease in the photosynthetic activity of the plant, together with an induction of the phenylpropanoid pathway and terpenoid biosynthesis [57]. Similar results were found in the *Vitis vinifera* infection by the same *Botrytis* species [65]. Also interesting is the infection by *Leptosphaeria maculans*, a damaging fungal pathogen that infects canola (*Brassica napus*), causing lesions on cotyledons and leaves, and cankers on the lower stem. Transcriptomic analysis of *L. maculans* infection in *Brassica* determined that the pathogen was able to activate the jasmonic acid and salicylic acid defense pathways in *B. napus*, consistent with defense against necrotrophs, with a coordinate shutdown of the photosynthesis genes [56]. In potato foliage and tubers, the oomycete *Pythopththora infestans* is able to produce serious infections with concomitant decrease of production. Using high-resolution transcriptomics by RNA-seq, Gao and coworkers demonstrated the existence of potato defense genes specifically expressed in tubers that are activated upon *P. infestans* infection [66]. Globally, and despite of the existence of some common responses against plant fungal pathogens, the majority of the differentially expressed genes upon infection are host-specific. In fact, in some specific cases, such expression differences may even underlie sex-specific responses of hosts to pathogen infections, most notably when pathogens induce partial sex reversal in infected hosts [67].

### 3.3. The Emerging New Roles of Non-Coding RNAs in Fungal Infections

Genome-wide analysis powered by the new next-generation sequencing technologies has revealed that eukaryotic genomes are extensively transcribed into thousands of long and short non-coding RNAs (ncRNAs). Those ncRNAs are key players in all cell functions, ranging from mitosis to signaling, and have also been related to some pathological processes, including the colonization of cells by infectious agents [68,69]. The role of two main classes of ncRNAs is starting to be investigated in the context of infection: micro-RNAs (miRNAs), an abundant class of short regulatory non-coding RNAs that act as post-transcriptional repressors by binding the 3’ UTR of target mRNAs [70]; and long intergenic non-coding RNAs (lincRNAs), a group of long RNA molecules (>200 nucleotides) that lack significant protein coding capacity and are involved in the regulation of gene expression at several cellular levels [71,72].

The expression profiles of non-coding RNAs, and more specifically of miRNAs, vary among different cell types, and the literature has presented evidence of the role of these ncRNAs in several human diseases, and more recent data also point to a critical role of miRNAs in fungal, bacterial, and viral infections [73,74,75]. Interestingly, intracellular infectious agents are usually able to induce a transcriptional program within the host cells that frequently includes an altered expression pattern of specific miRNAs [68,75,76]. The majority of the data already available supporting this evidence has been obtained by the analysis of viruses and bacteria, fungal infections being less studied. However, even isolated evidence obtained from well characterized fungal pathogens like *Candida* and *Aspergillus* indicate a clear relationship between the non-coding transcriptome and fungal infections.

In fungal infections, recent studies revealed that miR-155 plays an important role during maturation of dendritic cells, which inhibits the expression of the transcription factor PU.1 and thus decreases the levels of DC-SIGN, a fungal lectin able to recognize mannose-containing glycoproteins and the ability to bind *C. albicans* cells [77]. Furthermore, miR-155 can be of importance for various infectious diseases and may contribute to the susceptibility to infection and invasion by a range of pathogens [76]. Additionally, it was shown that addition of miR-155, miR-146, miR-146b, miR-455, and miR-125 can be regulated by bacterial lipopolysaccharide (LPS) and the toll-like receptor TLR4 [77]. *In silico* studies for miR-455 and miR-125a suggested that they may target some mRNA transcripts encoding proteins involved in signalling pathways of inflammatory response, suggesting that these miRNAs may also play a role in limiting inflammation. However, further studies are needed to delineate the role of these miRNAs in the innate immune system and its importance in response to fungal infection [77]. Recent evidence also demonstrated that miR-132 and miR-155 are differentially expressed in monocytes and dendritic cells upon stimulation with *A. fumigatus* or bacterial lipopolysaccharide (LPS), while miR-132 was induced only by *A. fumigatus*, suggesting that miR-132 could be a relevant regulator of the immune response directed specifically against this fungus [68].

The role of host miRNAs in fungal plant pathogens is also starting to be unveiled by taking advantage of specific RNA-seq techniques devoted to the analysis of small RNAs. Small RNA-seq is a variant of RNA-seq, which uses a preliminary enrichment step for the purification of RNAs of less than 200 nucleotides [78]. This approach has been recently applied for the analysis of the differentially expressed miRNAs in tomato upon infection by *P. infestans* [79], and in *Arabidopsis thaliana* infected with *F. oxysporum* [80].

In contrast, the role of lincRNAs as modulators of infection is still limited to a few descriptions in bacterial and viral infections. The first report of a long non-coding transcript associated with a bacterial infection was published by Cox *et al.* [81] in 2001. The authors described a previously uncharacterized lincRNA designated as HPYR1 (*Helicobacter pylori* Responsive Transcript 1), which was heavily upregulated in gastric mucosal cells infected with *H. pylori*. Similar infection-responsive lincRNAs have also been described in viral infections [82], but their functions in the modulation of the infection process are still unknown. However, the putative role of host lincRNAs in the context of fungal infections remains elusive and needs to be investigated.

Moreover, the role of the non-coding genome of the pathogenic fungi in the context of infection cannot be disregarded. In fact, fungal genomes contain a significant part of non-coding information, which can be transcribed into several families of ncRNAs [83]. Fungi are able to produce small miRNA-like molecules by using a primitive enzymatic system involved in RNA processing [84], and these molecules have been characterized in some pathogenic species such as *Penicillium marnefeii* [85], *Aspergillus flavus* [86] and *Cryptococcus neoformans* [87]. A very recent work by Peres da Silva and coworkers also described that several fungal species are able to secrete these small miRNA-like molecules to the extracellular medium, suggesting their possible roles in cell-to-cell communication [88]. Fungal genomes also contain transcriptional units responsible for the production of lincRNAs and their functional roles are starting to be unveiled. Recently published data showed the pivotal role of one of these fungal long RNAs, RZE1, in the context of morphological differentiation and virulence in *C. neoformans* [89].

## 4. High-Throughput Transcriptomic Methods for the Analysis of the Molecular Mechanisms Involved in the Interaction between Fungi and Host Cells

The interactions between a fungal pathogen and a host cell involve virulence factors produced by the pathogen together with the molecular response triggered by the infected cell. Both partners, pathogen and host, are characterized by a specific transcriptomic program which can be considered as a molecular fingerprint of their physiological state during their interactions. The use of high-throughput techniques for transcriptome analysis will allow for comprehensive monitoring of pathogen and host during their interaction [90].

Among high-throughput techniques for transcriptome profiling, the first introduced were hybridization microarrays. Microarrays rely on the hybridization of a labeled cDNA over a chip, where DNA probes are covalently immobilized, allowing the quantification of the expression levels of many genes simultaneously. Conceptually, this technique would be suitable for monitoring the gene expression of fungal pathogens and host cells during their interaction and has been applied successfully for the study of the transcriptomic changes of epithelial cells in response to *A. nidulans* infection [63]. However, there are severe limitations in this approach, mainly related to the need for pre-built microarrays with specific probes for the host and the pathogen, and also with the overall sensitivity of the method, which prevents its applications for infections with a low level of colonization of the host cell.

**Figure 1:**
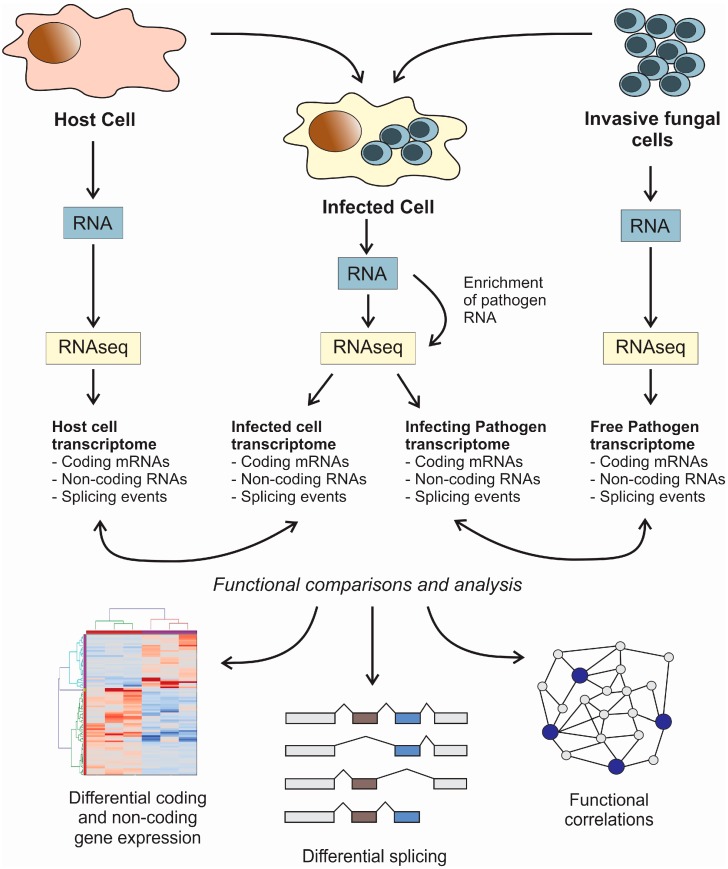
Flowchart for the dual RNA-seq protocol for simultaneous transcriptomic analysis of fungal pathogens and host cells. The represented scheme can be applied in steady-state and time-course experiments. Note that in order to increase the sensitivity of the method, it would be advisable to perform an enrichment of the fungal pathogen RNA when the infected cell is analyzed. This enrichment can be performed by microarrays or biotinylated probes. In the particular case of the study of small ncRNAs such as miRNAs (small RNA-seq), an additional step must be performed in order to purify the small RNAs before library preparation. Functional comparisons between samples will allow for study of the differentially expressed coding and non-coding genes and the alternative splicing events.

The application of next-generation sequencing (NGS) methods for the simultaneous study of host and pathogen transcriptomes during their interaction have been designated as “dual RNA-seq” [90]. NGS has important advantages over microarrays, since it is a probe-independent method that can be easily adapted to any possible pathogen and host, it is comparatively cheaper, and offers an increased sensitivity and discriminatory power [90,91]. The subjacent idea in the application of the method is to simultaneously sequence RNA belonging to a mixed population of host and pathogen cells. RNA reads coming from each cell can be distinguished by alignment with the corresponding genomes (Figure 1) [57]. Existing NGS platforms for high-throughput sequencing are very diverse, and their technical characteristics, advantages, and drawbacks were already reviewed elsewhere [92]. The main advantage of dual RNA-seq is that it is an unbiased approach, which can be used not only to detect differentially expressed genes, but also to pinpoint dynamic changes in transcriptional regulatory events such as alternative splicing. This fact is especially relevant when we are dealing with eukaryotic pathogens such as fungi. Dual RNA-seq can be performed in a steady state version not only for analyzing infected *vs*. non-infected systems but it is also suitable for application to time-course studies, where a fungal infection can be followed over time [64]. Time-resolved analysis will often require a dedicated data analysis strategy, where gene expression data must be combined with cellular pathways in a holistic fashion to infer inter-species regulatory networks [57]. Several computer applications have been designed with the objective of analyzing dual RNA-seq data in different contexts. Among them, RNA CoMPASS is a user-friendly RNA-seq pipeline for the analysis of transcriptomes from diverse biological specimens that can be adapted to dual RNA-seq data [93]. Other applications, such as NetGenerator, are specifically designed to analyze time-resolved multi-stimuli and multi-experiment data, and are extremely useful to infer cross-regulatory networks from dual RNA-seq data [94,95].

However, one of the most prominent drawbacks of any method to perform a simultaneous analysis of host and pathogen transcriptomes is the relatively low abundance of transcripts belonging to the pathogenic organism. This is particularly relevant when we deal with invasive fungi, since their colonization abilities are extremely variable [11]. Estimated data obtained from the analysis of several invasive pathogens, including bacteria, showed that the expected amount of RNA coming from the pathogen in a dual RNA-seq experiment is between one and five percent [90]. To overcome this limitation, several methods for selective enrichment of RNAs from the colonizing pathogen have been described. Among them, SAGE [96] and SuperSAGE [61] have been shown to be useful in the detection and quantification by dual RNA-seq of RNAs from the pathogenic microorganism [61]. Enrichment protocols are based on the selective capture of RNAs from the pathogenic microorganism using specific probes that can be immobilized in a microarray chip or biotinylated and further captured in streptavidin beads. This enrichment strategy has been successfully employed in the dynamic characterization of transcriptomic crosstalk of host and pathogen during *C. albicans* infection using mouse and insect models [30].

## 5. Conclusions

The phenotypic change observed in a host cell when it is infected by a fungal pathogen is a direct consequence of specific transcriptional programs induced by the pathogen. Although different pathogens tend to take advantage of similar pathways in the host, the methods used to hijack the host cell are usually pathogen-specific [97]. Fungal pathogens are unique in their virulence characteristics and abilities to colonize the host cells, and are comparatively less studied than bacteria. The accumulated experimental evidences in the last decade clearly showed distinct transcriptional patterns in host cells in response to fungal infections [68,91]. However, the functions and roles of the differentially expressed coding and non-coding genes in the context of infection remain elusive.

High throughput transcriptomic analysis empowered by next-generation sequencing technologies has become an important resource of information for the detailed molecular analysis of the interaction between fungal pathogens and their hosts. However, the use of this technique in the field of fungal infections is still limited to a few examples in model organisms [30,63]. Despite the development of new methods of analysis of next-generation sequencing data [93], there is an urgent need to obtain more data in different fungal pathogens and hosts to dissect the mechanism of infection, taking into account common and specific facts among fungal pathogens. New players in this interaction are also starting to be unraveled, such as epigenetic factors including chromatin modifying enzymes and non-coding RNAs [98,99].
